# Effects of Neuromuscular Training on Stable Versus Unstable Surfaces on Unilateral Force Production and Stability in Elite Male Soccer Players

**DOI:** 10.3390/jfmk10040379

**Published:** 2025-10-01

**Authors:** Sergio Jiménez-Rubio, David García-Albín, José Luis Estévez Rodríguez, Sergio L. Jiménez-Sáiz

**Affiliations:** 1 Sport Sciences Research Centre, Rey Juan Carlos University, 28943 Fuenlabrada, Spain; sergio.jimenez.rubio@urjc.es; 2 Joint Group on Frailty and Successful Aging UCLM-SESCAM, University of Castilla-La Mancha-Health Service of Castilla-La Mancha, IDISCAM, 45002 Toledo, Spain; david.garcia129@alu.uclm.es; 3 GENUD Toledo Research Group, Faculty of Sport Sciences, University of Castilla-La Mancha, 45071 Toledo, Spain; 4 Switzerland National Team, Worbstrasse 48, 3074 Muri bei Bern, Switzerland; joselu.estevez@gmail.com; 5 Faculty of Physical Activity and Sport Sciences, INEF-Sports Department, Polytechnic University of Madrid, 28040 Madrid, Spain

**Keywords:** proprioception, ankle injuries, neuromuscular control, stability

## Abstract

**Background:** Neuromuscular training is widely implemented in professional football to enhance performance and reduce injury risk. Although unstable surfaces are commonly used for proprioceptive and rehabilitation purposes, limited evidence supports their effectiveness in improving sport-specific force production and stability in elite athletes. This study aimed to compare the effects of multicomponent neuromuscular training performed on stable versus unstable surfaces on unilateral force production, mobility, and agility in elite male soccer players. **Methods:** Twenty-seven professional male soccer players from the Spanish first division were randomly assigned to either a stable surface group (SSG; *n* = 14) or an unstable surface group (USG; *n* = 13). Both groups completed a 10-week intervention in addition to their regular training routines. Pre- and post-intervention assessments included dorsiflexion range of motion (DFt), Y-Balance Test (YBT), single-leg countermovement jump (SLCMJ), single-leg hop for distance (SLH), side-hop (SH), Speedy Jump (SpJ), Agility T-test (TT), and the Lower Extremity Functional Test (LEFT). A two-way repeated-measures ANOVA and Hedges’ g effect sizes were used for statistical analysis. **Results:** The SSG showed significant improvements in most performance variables, including DFt, YBT, SLH, SH, SpJ, TT, and LEFT (percent change range: 1.6% to 9.8%; Hedges’ g ranging from 0.52 to 2.57). The USG showed limited improvements, with significant changes only in LEFT (percent change = 1.18%; Hedges’ g = 0.53). Notably, the stable surface group demonstrated enhanced force production and agility, particularly in the non-dominant limb. **Conclusions:** Multicomponent neuromuscular training on stable surfaces appears more effective than training on unstable surfaces for improving unilateral strength, mobility, and agility in elite soccer players. These findings suggest that stable surface training may provide superior performance benefits and should be considered a priority in high-performance environments.

## 1. Introduction

The demanding schedules in professional football necessitate the optimisation of training programmes to improve player performance, ensure optimal recovery, minimise injury risk, and maximise player availability for training and competition [1,2]. High-intensity actions are inherent to football and represent key determinants of performance. Movements such as sprinting, jumping, changing direction, and shooting require high levels of strength and power output from the neuromuscular system [3,4]. A recent meta-analysis has concluded that strength training plays a fundamental role in injury prevention, not only improving performance, fitness level, speed and agility, but also improving the recovery process after an injury [5]. Many of the most decisive actions in football are executed unilaterally, relying on single-leg force production. It is also noteworthy that a large proportion of injuries occur in single-leg support phases [6,7]. Kuranganti et al. [8] defined bilateral limb deficit (BLD) as a phenomenon that refers to the difference in the ability to generate maximum force depending on whether muscles are contracted individually or together with muscles on the opposite side of the body. This deficit manifests itself when the sum of the forces generated unilaterally exceeds the force produced during a bilateral contraction. Inter-limb asymmetries have been identified as potential indicators of lower limb injury risk [9]. These imbalances can be detected through differences in mobility, strength, and power between limbs. Notably, unilateral strength training has been shown to reduce these asymmetries and improve performance in different tests [10]. A recent meta-analysis [11] compared unilateral versus bilateral plyometric training and found that unilateral protocols significantly enhanced jump height, sprint speed, and change-of-direction ability. In contrast, bilateral training primarily improved bilateral jump performance. Unilateral training is considered more sport-specific due to its similarity to the movement patterns observed in team sports [12,13]. As a result, ongoing research seeks to identify and validate training strategies that offer evidence-based support for coaches aiming to design efficient and sport-specific conditioning programmes [3,11].

The emergence of new methodologies and concepts surrounding training has generated considerable controversy when it comes to defining training programmes. In recent years, different forms of training have appeared that have been defined as functional training, in which elements of instability and new training elements are used, but no significant results have been found in relation to the benefits they generate on the athlete’s performance [14]. In certain sport-specific scenarios, athletes must perform under unstable conditions. Therefore, training should attempt to replicate the demands of the sport as closely as possible, aligning with the principle of specificity [15]. Although unstable surface training has traditionally been used in rehabilitation settings, it has also been integrated into strength training programmes to challenge postural control, enhance core stability, and improve proprioceptive neuromuscular feedback. These adaptations aim to develop stability and balance in athletic movements. [16,17]. However, several studies have reported that instability conditions can negatively impact lower limb biomechanics during jumping and landing tasks [18,19]. For instance, individuals with chronic ankle instability demonstrate reduced activation of lumbo-pelvic stabilisers and the peroneus longus during landing, along with increased ankle inversion and plantarflexion—factors that elevate the risk of lateral ankle sprains [18]. Moreover, unstable landings have been associated with elevated ground reaction forces and altered joint kinematics, including increased knee valgus and reduced flexion angles, which may lead to greater stress on the anterior cruciate ligament (ACL) and a heightened risk of non-contact ACL injuries [16].

To date, no studies have explored the effects of unilateral instability training on force production and sport-specific performance in professional football. Therefore, the present study aimed to evaluate the effects of a 10-week unilateral training programme performed on stable versus unstable surfaces on jumping, sprinting, change-of-direction ability, and mobility, as well as on neuromuscular control in single-leg stance. We hypothesise that training performed on stable surfaces will result in greater performance improvements related to force production in multiple movement planes.

## 2. Method

### 2.1. Study Design

A prospective experimental study was designed to investigate the effects of two different preventive and conditioning training programmes, one of them based on stability and force production tasks on stable surfaces and the other involving similar tasks performed on unstable surfaces. Pre- and post-training assessments included the Dorsiflexion Test (DFt), Y-balance test (YBT), single leg countermovement jump (SLCMJ), single leg hop for distance test (SLH), side-hop test (SH), speedy jump test (SpJ). All tests were conducted in the morning (9–11 a.m.) on the team’s regular natural grass field, with ambient conditions of 17–22°C and 60–70% humidity. The assessment sessions were supervised by the same strength and conditioning specialist, and players were instructed not to eat without the nutritionist’s advice in the 24 h prior to the tests.

### 2.2. Participants

**Figure 1:**
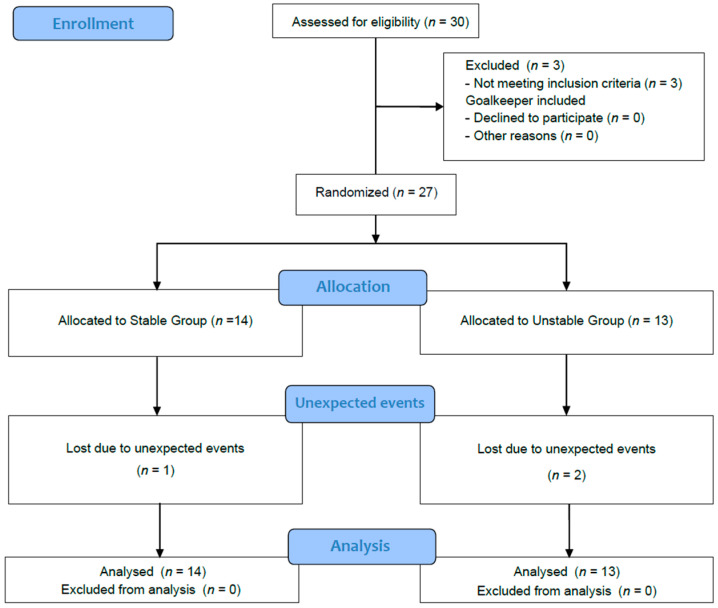
Flow Chart.

Initially, a total of thirty Spanish male professional soccer players (age: 24.2 ± 2.8 years, height: 179.3 ± 2.8 cm, body mass: 69.1 ± 2.4 kg, body mass index: 21.1 ± 0.8 kg·m^−2^) voluntarily participated in the study. An a priori power analysis was performed (G*Power, v3.1.9.2, Universität Kiel, Kiel, Germany), which determined that a sample size of at least 20 participants was needed to achieve a power (1-β) of 0.84, assuming an effect size (ES) of 0.35 (moderate effect) and alpha of 0.05. All participants played for the same professional football team competing in the Spanish first division (LaLiga) during the 2021/2022 season. On average, players trained 7.1 ± 0.82 h per week and participated in one match per week during the season. The inclusion criteria for the study were as follows: professional footballers over the age of 18 with no history of cardiovascular or metabolic diseases, able to perform stability exercises on one leg and without injuries during the research process. Players with joint injuries in the two months prior to the study and with a recent history (i.e., less than ten weeks) of knee surgery were excluded from the study.Goalkeepers were excluded from the statistical analysis due to the different physical demands placed on them in football. Participants were randomly assigned to either the stable surfaces group (SSG, *n* = 14) or to the unstable surfaces group (USG, *n* = 13). Finally, 27 soccer players (i.e., SSG, *n* = 14 and USG, *n* = 13) were included in the further analysis since two participants left the club during the winter transfer market and one more was injured during the assessment sessions (Figure 1). All soccer players were provided with detailed information about the study procedures, potential risks, and benefits, before they signed a written informed consent form. The study was in accordance with the Declaration of Helsinki (2013), and the data collection procedure was approved by the ethical committee of the Universidad Rey Juan Carlos (Madrid) with internal registration number 3105202214522, on 20 July 2022.

### 2.3. Procedures

**Figure 2:**
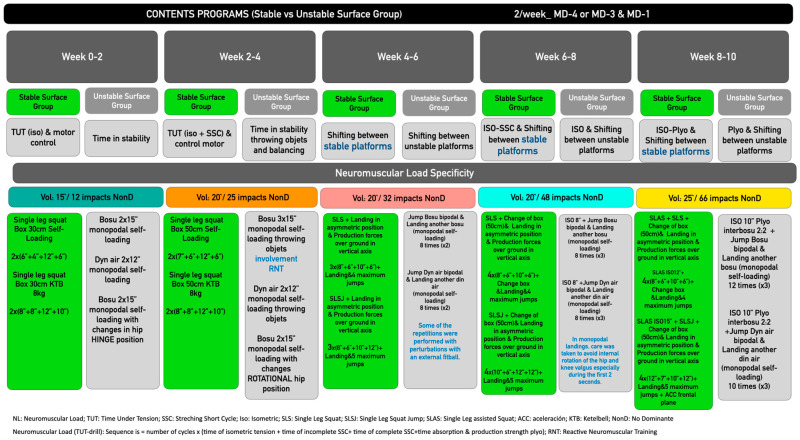
Multicomponent Training Program Stable vs. Unstable.

During the 10-week intervention period, players performed their regular weekly in-season routine (Table 1), with the SSG performing their preventive program 3–4 times per week, in addition to their regular soccer training routines. Both groups performed a different joint and muscle injury risk prevention programme, based on neuromuscular control and the ability to produce unipodal strength, some on stable surfaces and others mainly on unstable surfaces (Figure 2).

All participants were familiar with the testing protocols due to the club’s routine practices. All fitness tests were performed in a single session in the following order: DFt, YBT, SLCMJ, SLH, SH, SpJ, TT, and LEFT. Previous studies have used similar tests [19]. Before the test, the players performed a standardised 12-min warm-up consisting of four blocks: (a) normalisation of myofascial tissue, in which participants used foam rollers and pressure balls to optimise the myofascial level in the quadriceps, soleus and hamstrings [20]; (b) mobility, facilitating the range of motion of the thoracic spine, the ankle in dorsiflexion, the hip in submaximal flexion combined with external rotation, overhead squat, and analytical facilitation of internal hip rotation; (c) muscle activation, based on the application of tension to the posterior chain with three exercises combining isometrics and short cycles of stretching-shortening on safe levers; and (d) adaptation to the stretch-shortening cycle, applying the one-leg jump exercise with take-offs approximately 2 cm above the surface and the counter-movement jump without seeking maximum height, but rather motor control in the deceleration phase.

#### 2.3.1. Dorsiflexion Test (DFt)

The ankle dorsiflexion test is a necessary measure to evaluate the mobility of the tibioperoneoastotalar joint. The ankle joint needs to have an optimal range of motion to be effective in movement and subsequent force production/absorption. For this, it is advisable to follow the joint by joint guidelines described in some studies [21].

About the assessment, some studies [22] have evaluated this variable in cm according to the protocol of Calatayud et al. 2015 [23]. We also found in the literature works that have compared the use of different devices to obtain the range of joint motion in the ankle, inertial (Wimu^TM^), standard goniometer and 2D video analysis with Kinovea® [24]. In the present work, the Kinovea® application version 0.8.15 was used to obtain the value of the dorsiflexion in degrees [25,26].

The test consisted of evaluating the degrees of the joint while moving the tibia forward, without separating the calcaneus from the ground, and avoiding bringing the hip into internal rotation or collapse of the medial longitudinal arch. The intraclass correlation coefficients (ICCs) and the coefficients of variation (CVs) for the DFtd were 0.81 (0.63–0.9) and 3.9% (3.4–5.1) and ICC = 0.87 (0.65–0.93) for DFtd, and 4.1% (3.8–5.5) and 0.89 (0.68–0.89) for DFtnd.

#### 2.3.2. Y-Balance Test (YBT)

The players performed two attempts with each leg, the dominant leg (YBTd) and the non-dominant leg (YBTnd), with 60 s of passive recovery. The Y balance test kit (Move2Perform®, Evansville, IN, USA) was used for this purpose. Participants began in a standing position on one leg, with the toes of the supporting foot placed on the red line marked on the central platform of the instrument. The players then began the test by pushing with the toes of the contralateral foot as far as possible in three directions: anterior, posteromedial, and posterolateral. For correct execution, the supporting leg maintained full contact with the platform, while the contralateral leg maintained constant contact with the sliding elements. The players then returned to the starting position and held a final balance position on one leg for two s for the test to be considered valid. To normalise the scores, limb length was measured and performance was calculated using the following formula: composite score = (anterior + posteromedial + posterolateral performance in cm)/3 × limb length in cm) × 100 [27]. The best attempt, defined as the highest recorded value, was selected for further analysis. The ICCs and CVs for the YBT were 0.87 (0.70–0.93) and 1.8% (1.5–2.5) for YBTd, and 0.92 (0.82–0.97) and 1.4% (1.3–2.0) for YBTnd.

#### 2.3.3. Single Leg Countermovement Jump (SLCMJ)

The footballers performed two attempts with each leg, the dominant (SLCMJd) and the non-dominant (SLCMJnd), with a passive rest period of 35 s between them. The height of the jump was measured using a photocell system (Optojump, MicrogateTM, Bolzano, Italy). It was calculated using the formula: h = gt/8 (h, height, cm; g, acceleration due to gravity, 9.81 m·s^−2^; t, flight time of the jump, s) [28]. The players started in a standing position on one leg and from there performed a triple flexion with the supporting leg. From that point, they jumped as high as possible [29]. In addition, the players were randomised to start the test with a different leg. The highest jump height (cm) for each leg was selected for further analysis. The ICCs and CVs for the SLCMJ test were 0.82 (0.67–0.91) and 4.1% (3.2–5.3) for SLCMJd, and 0.85 (0.62–0.91) and 4.6% (3.7–6.2) for SLCMJnd.

#### 2.3.4. Single Leg Hop for Distance Test (SLH)

The players completed two attempts at the test with each leg, the dominant (SLHd) and the non-dominant (SLHnd), using a tape measure to measure the distance parameter. This was performed with a passive recovery period of 35 s between attempts. The players started on one leg, positioned on a starting line marked on a fixed signal. They then jumped as far as possible, landing on the same leg. For the attempt to be considered valid, the landing had to be stable for two s. The distance was measured in centimetres, from the starting line of the mark (take-off point) to the heel of the participants where the landing occurred [30]. The attempt that resulted in the greatest distance was recorded for further analysis. The ICCs and CVs for the SLH test were 0.98 (0.96–0.98) and 1.1% (1.0–1.6) for SLHd, and 0.98 (0.96–0.98) and 1.1% (0.8–1.3) for SLHnd.

#### 2.3.5. Limb Simetry Index for SLCMJ (LSI-SLCMJ)

The limb symmetry index for the single-leg countermovement jump (LSI-SLCMJ) was calculated from the best attempt of each leg (SLCMJd and SLCMJnd). The index was obtained using the following formula: LSI (%) = (min[SLCMJd, SLCMJnd]/max[SLCMJd, SLCMJnd]) × 100, so that a value of 100% indicates perfect inter-limb symmetry and lower values reflect greater asymmetry.

#### 2.3.6. Side-Hop Test (SH)

Football players performed two trials with each leg, the dominant (SHd) and the non-dominant (SHnd), with a passive recovery period of 80 s between them. Participants were instructed to complete as many jumps as possible over 30 s, which was measured with a stopwatch. Starting from a standing position on one leg with hands on the hips, players had to jump laterally across two parallel lines, 35 cm apart, drawn on the therapy mat. After the final jump, they were required to maintain a controlled landing for two s, also timed with a stopwatch. Jumps that did not meet the controlled landing criteria were considered errors and subtracted from the total number of jumps [30]. For subsequent analysis, the trial with the highest number of correct jumps (score = total jumps − erroneous jumps) was selected. The ICCs and CVs for the SLH test were 0.93 (0.91–0.96) and 1.4% (1.1–2.3) for SHd, and 0.89 (0.81–0.94) and 2.0% (1.5–2.6) for SHnd.2.3.7. Speedy jump test (SpJ).

The footballers performed two attempts at the speed jump test (SpJ) with each leg, the dominant (SpJd) and the non-dominant (SpJnd), with 85 s of passive recovery between them. Each attempt consisted of three jumps over each of the four red bars (i.e., jumping forward, backward, and forward) and one jump over each of the four blue bars (jumping sideways), for a total of sixteen jumps [31]. For this test, a predetermined set of basic rapid jumps (TST, Trendsport®, Grosshöflein, Austria) was used. Participants started in a standing position on one leg and performed the jumps according to the specified sequence. After the last jump, a controlled landing on the same leg had to be maintained for three s, measured with a stopwatch. If the landing was not stable for the required time, the attempt was marked as invalid. The test execution time was measured in seconds from the moment of the first jump (take-off phase) to the moment of the last jump (landing). The attempt with the shortest execution time was taken into account for further analysis. The ICCs and CVs for the SpJ test were 0.71 (0.62–0.87) and 4.3% (3.2–6) for SpJd, and 0.73 (0.61–0.89) and 4% (3.1–5.3) for SpJnd.

#### 2.3.7. Agility T-Test (TT)

Two attempts at the test were completed with a passive rest period of 80 s between them, remaining standing during this period. A photocell system (Polifemo, MicrogateTM, Bolzano, Italy) was used to measure the time required to complete the test. The players followed the previous protocol [32]. The fastest attempt, determined by the recorded time, was used for subsequent analysis. The ICC and CV for the TT were 0.74 (0.48–0.85) and 5.1% (4.4–6.6).

#### 2.3.8. Lower Extremity Functional Test (LEFT)

The footballers performed two attempts at the test with a passive rest period of 90 s between them. Protocol [33] was used to perform this test. The test consists of 16 specific manoeuvres, including forward and backward sprints, side steps, cross steps, and 45- and 90-degree cuts, which the players must complete as quickly as possible. A photocell system (Polifemo, MicrogateTM, Bolzano, Italy) was used to measure the time taken to complete the test. The best attempt, determined by the shortest time taken to complete the test, was considered for further analysis. The ICC and CV for the LEFT were 0.81 (0.64–0.9) and 1.3% (1.1–1.5).

### 2.4. Multicomponent Training Programs

The training interventions in both groups lasted 10 weeks, standardizing the frequency of training sessions with 2 weekly sessions in MD-4 or MD-3 for the first intervention and MD-1 for the second weekly intervention. In both groups, players were introduced to the strength and stability exercises during the pre-season period, which allowed them to familiarise themselves with the programmes. Each training session lasted approximately 20–30 min (Figure 2) and took place immediately after a specific warm-up routine, prior to the regular football training sessions (75–90 min). The indoor intervention sessions were supervised by the team’s strength and conditioning staff, who provided adequate information and instructions to execute the exercises correctly. In each of the programmes, to ensure training principles, a progressive overload approach was applied, adapting the workload for each exercise according to individual capabilities. Each programme applied specific details of landings, relationship between force production-absorption, application of force across multiple vectors (Figure 2).

### 2.5. Statistical Analysis

All statistical analyses were conducted using SPSS Statistics version 29.0.1 (IBM Corp., Armonk, NY, USA). The analyses followed a per-protocol approach, excluding the three participants who did not meet the inclusion criteria. Data are expressed as mean ± standard deviation (SD) for each intervention group. The Shapiro–Wilk test was used to assess normality; all variables followed a normal distribution (*p* > 0.05). Parametric methods were used to examine differences between groups. A two-way analysis of variance (ANOVA; 2X2, corresponding to group [Stable vs. Unstable] × time [Pre vs. Post]) was used to compare the effect of each intervention and their interaction. When a significant F-statistic was observed for any main or interaction effect, Bonferroni-adjusted post hoc comparisons were conducted to explore pairwise differences within groups (i.e., pre- to post-intervention changes) and between groups (i.e., comparisons between groups at pre- and post-assessments). Effect sizes for pre- to post-intervention comparisons were calculated using Cohen’s d for paired samples, with Hedges’ correction applied for small sample bias. Effect sizes were reported as Hedges’ g. The magnitude of the effect size was interpreted as follows: trivial (d = 0–0.19), small (d = 0.20–0.49), medium (d = 0.50–0.79), and large (d ≥ 0.80). Statistical significance was set at *p* < 0.05 for all analyses.

## 3. Results

Table 1 presents the main effects of time, group, and the time × group interaction for each dependent variable, as derived from the two-way ANOVA. Statistical outputs, including F-statistics, *p*-values, and partial eta squared (η^2^p), are presented in Table 1. Table 2 provides the pre- and post-intervention means, standard errors, and within-group effect sizes (Cohen’s d with Hedges’ correction).

Significant main effects of time and time × group interactions were identified for DFt. Moderate effect sizes were observed (Hedges’ g = 0.74 for DFTd and Hedges’ g = 0.77 for DFTnd) in the stable group. No significant pre-post changes were found in the unstable group.

For YBT, significant time effects and group*time interactions were observed. Large effect sizes were found (Hedges’ g = 2.45 for YBTd and Hedges’ g = 2.57 for YBTnd) in the stable group. No significant pre-post changes were detected in the unstable group.

In SLCMJ, significant main effects of group and time were detected, as well as a group × time interaction in the non-dominant leg. Large effect sizes were found (Hedges’ g = 1.14 for SLCMJd and Hedges’ g = 1.36 for SLCMJnd) in the stable group. No significant changes were observed in the unstable group.

In LSI SLCMJ, significant main effects of group and a group × time interaction were detected, whereas no main effect of time was observed. The stable group showed a significant decrease in LSI from pre to post (91.1 ± 1.3% to 87.2 ± 0.7%; *p* = 0.010; g = –0.79), indicating increased inter-limb asymmetry, while the unstable group remained unchanged (96.9 ± 1.3% to 97.9 ± 0.8%; *p* = 0.524; g = 0.15).

For SLH, significant main effects of time and group*time interactions were found. Effect sizes ranged from small to large (Hedges’ g = 0.17 for SLHopd and Hedges’ g = 1.02 for SLHopnd) in the stable group. No significant pre-post changes were detected in the unstable group.

In SH performance, significant time effects and group*time interactions were observed. Moderate to large effect sizes were found (Hedges’ g = 0.52 for SHd and Hedges’ g = 1.19 for SHNnd) in the stable group. No significant changes were observed for SHd in the unstable group. However, a moderate negative effect size (Hedges’ g = −0.60) was identified for SHNd, indicating a statistically significant performance decline.

For SpJ, significant time effects were observed. Moderate to large effect sizes were found (Hedges’ g = −1.06 for SpJd and Hedges’ g = −0.68 for SpJnd) in the stable group. No significant changes were detected in the unstable group.

Significant main effects of time and significant time × group interactions were observed for both TT and LEFT. For TT, a large effect size (Hedges’ g = 0.80) was observed in the stable group. No significant change was detected in the unstable group.

For the LEFT, a large negative effect size (Hedges’ g = −1.02) was found in the stable group, while the unstable group exhibited a moderate positive effect (Hedges’ g = 0.53).

## 4. Discussion

This study proposes a novel approach to training on different surfaces for both injury prevention and performance optimisation in professional football. It compares multicomponent training programmes conducted on stable and unstable surfaces, targeting key physical capacities such as stability, jumping, and agility—all fundamental components of football performance.

The main findings revealed that the training programme performed on stable surfaces had a greater positive impact on performance-related variables compared to the unstable surface intervention.

Football is a multidirectional team sport that requires players to repeatedly execute jumps across different planes of movement [34], often under unpredictable conditions [35]. After the 10-week intervention, players in the stable surface group showed improvements in jumping performance across nearly all assessments (i.e., DF, SLCMJ, SLH, SH, and SpJ for both limbs), except for SLCMJd. These improvements may be related to faster activation of motor units, which facilitates faster and more coordinated muscle responses. [36]. These findings are consistent with previous research [37], which reported improved multidirectional jump performance in youth footballers following unilateral, core-focused, and jump-based multicomponent training.

However, unlike other unilateral tests in which the dominant and non-dominant limbs responded similarly or without significant changes between groups [38], the results of the SLCMJ variable showed a divergent adaptation pattern between the legs, with an increase in performance in the non-dominant limb and a decrease in the dominant limb. In order to obtain the overall effect of these op-posing changes, a limb symmetry index (LSI SLCMJ) was calculated. Therefore, the symmetry analysis was limited to the SLCMJ, as this test, as seen in recent litera-ture [39], is one of the most relevant and sensitive parameters for assessing limb imbalances.

The divergent adaptations observed in the SLCMJ can be explained in part by the greater margin for improvement in the non-dominant limb, which allowed for more pronounced gains compared to the dominant side. Conversely, the dominant limb, which is usually more involved in specific football actions such as kicking, may have shown a lesser adaptive response during the intervention. In addition, the non-dominant limb is the one used for propulsion and stabilisation within the game.

Furthermore, it is known from previous studies [40] that jump-based assessments are intrinsically influenced by variability between trials and technical execution, and therefore we consider that small fluctuations in athletes’ performance results cannot be completely ruled out.

It is worth noting that the unstable group obtained favourable results in the speed and agility variable (LEFT). We reviewed studies in which other subjects increased their scores in similar tests [41] and found that the reduction in execution time after training on unstable surfaces could be related to these effects. Other studies also justify changes in this agility test between healthy and injured players [42].

Although unstable surfaces have traditionally been used in rehabilitation settings to improve proprioception [43], our results indicate that training on stable surfaces provides greater benefits in enhancing ankle dorsiflexion (particularly in the tibioper-oneal joint), multiplanar stability, and unilateral force production—especially in the non-dominant limb. In contrast, the unstable group only showed significant improve-ments in the SHnd and LEFTs, with no relevant gains in vertical-horizontal force production, stability, or ankle mobility.

Furthermore, the stable group’s superior performance in change-of-direction and agility tests reinforces the value of stable surface training for neuromuscular adaptation and motor control. Recent evidence [44] supports this notion, highlighting the role of targeted force production strategies in enhancing agility and athletic performance among elite athletes.

Both training programmes followed structured progression criteria throughout their mesocycles. Notably, the increase in the number of landings over time may explain the improvements observed in variables such as SLCMJ, SLH, and Speedy Jump.

The adaptations observed in the stable surface group may be attributed to the specific design of the final training phases (weeks 6–10), which incorporated isometric–plyometric combinations, reduced ground contact time, and reactive multidirectional movement patterns. This approach aligns with previous findings [45], in which similar training elements were associated with performance gains.

Future studies should investigate limb dominance more thoroughly to better understand inter-limb adaptation patterns. Based on the present results, stable surface training is recommended as a primary strategy for enhancing strength, agility, and reactive performance in elite football players.

### Practical Applications

The results of this study support the integration of stable-surface neuromuscular training as a key component of in-season injury prevention and performance enhancement programmes in elite football. Practitioners are advised to prioritise unilateral exercises on stable surfaces to improve ankle dorsiflexion mobility, single-leg force production, and multidirectional stability—particularly in the non-dominant limb. Furthermore, the inclusion of progressive overload and the reduction in ground contact time during weeks 6 to 10 may further augment the training effects. These findings provide practical guidance for strength and conditioning professionals aiming to develop effective and context-specific training strategies.

## 5. Conclusions

The findings of this study demonstrate that neuromuscular training performed on stable surfaces is more effective than unstable-surface training for enhancing physical performance in elite football players. This type of training appears to be more cost-efficient, transferable to sport-specific tasks, and conducive to performance optimisation in high-demand competitive environments. Accordingly, structured and controlled stable-surface training is recommended to improve unilateral strength, mobility, and agility, ultimately enhancing readiness for multidirectional actions during match play.

## Figures and Tables

**Table 1:** Two-way repeated-measures ANOVA results for each variable. F-values, *p*-values, and partial eta squared (η^2^p) shown for main effects of group, time, and the group × time interaction.

		Group		Time		Interaction Group × Time		
		F	*p*	F	*p*	F	*p*	η2p
**DFt**	DFtd	10.09	0.044 *	8.16	0.009 *	16.36	<0.001 *	0.39
	DFtnd	5.81	0.024 *	5.67	0.025 *	6.901	0.015 *	0.22
**YBT**	YBTd	26.80	<0.001 *	10.03	0.004 *	20.75	<0.001 *	0.45
	YBTnd	50.68	<0.001 *	66.54	<0.001 *	81.36	<0.001 *	0.76
**SLCMJ**	SLCMJd	41.79	<0.001 *	9.29	0.005 *	4.53	0.432	0.15
	SLCMJnd	54.20	<0.001 *	8.24	0.008 *	17.40	<0.001 *	0.41
**LSI SLCMJ**		45.02	<0.001 *	2.18	0.152	5.79	**0.024**	0.18
**SLH**	SLHopd	1.83	0.188	0.59	0.448	7.75	0.010 *	0.24
	SLHopnd	3.78	0.063	39.72	<0.001 *	41.39	<0.001 *	0.62
**SH**	SHd	26.95	<0.001 *	0.16	0.691	9.63	0.005 *	0.28
	SHNnd	71.17	<0.001 *	0.64	0.429	25.49	<0.001 *	0.50
**SpJ**	SpeedJd	2.19	0.151	35.45	<0.001 *	24.12	<0.001 *	0.49
	Speednd	3.63	0.068	29.09	<0.001 *	21.98	<0.001 *	0.47
**TT**		0.80	0.379	3.80	0.622	32.71	<0.010 *	0.13
**LEFT**		3.71	0.065	6.36	0.018 *	52.50	<0.010 *	0.67

**Notes.** d and nd indicate dominant and non-dominant limbs, respectively. DFt = dorsiflexion test; YBT = Y-balance test; SLCMJ = single-leg countermovement jump; SLH = single-leg hop for distance; SH = side-hop test; SpJ = speed jump test; TT = agility T-test; LEFT = lower extremity functional test. SE = standard error. Statistical significance set at *p* < 0.050; * indicates statistically significant differences in comparisons in factor or interaction inter-groups.

**Table 2:** Pre- and post-intervention means ± standard deviation (SD) and within-group effect sizes (Hedges’ g) for each variable by group.

		Unstable Group				Stable Group			
Variables		Pre	Post	*p*	*d*	Pre	Post	*p*	*d*
**DFt**	DFTd	29.8 ± 0.6	29.5 ± 0.5	0.411	−0.16	31.2 ± 0.6	33.0 ± 0.5	<0.001 *	0.74
	DFTnd	30.3 ± 0.5	30.2 ± 0.5	0.861	−0.04	31.0 ± 0.5	32.6 ± 0.5	0.001 *	0.77
**YBT**	YBTd	84.4 ± 0.7	84.0 ± 0.7	0.338	−0.11	88.3 ± 0.7	90.2 ± 0.6	<0.001 *	0.76
	YBTnd	83.2 ± 0.7	82.9 ± 0.7	0.543	−0.10	86.5 ± 0.7	92.5 ± 0.6	<0.001 *	2.57
**SLCMJ**	SLCMJd	15.4 ± 0.3	15.2 ± 2	0.524	−0.20	17.9 ± 0.3	16.7 ± 0.2	<0.001 *	−1.14
	SLCMJNnd	15.8 ± 0.3	15.5 ± 0.2	0.372	−0.28	17.4 ± 0.3	19.1 ± 0.2	<0.001 *	1.36
**LSI SLCMJ**		96.9 ± 1.3	97.9 ± 0.8	0.524	0.48	91.1 ± 1.3	87.2 ± 0.7	**0.010**	−0.67
**SLH**	SLHopd	131.2 ± 3.1	130.0 ± 2.5	0.170	−0.13	134.8 ± 2.9	136.9 ± 2.4	0.010 *	0.17
	SLHopnd	129.4 ± 2.4	129.3 ± 1.9	0.923	−0.01	130.2 ± 2.3	139.9 ± 1.8	<0.001 *	1.02
**SH**	SHd	51.4 ± 0.7	50.6 ± 0.6	0.721	−0.29	54.8 ± 0.6	55.9 ± 0.6	0.010 *	0.52
	SHnd	52.6 ± 0.6	50.9 ± 0.6	0.007 *	−0.60	57.5 ± 0.6	59.9 ± 0.6	<0.001 *	1.19
**SpJ**	SpeedJd	7.6 ± 0.1	7.5 ± 0.1	0.473	−0.15	8.2 ± 0.1	7.5 ± 0.1	<0.001 *	−1.06
	Speednd	7.6 ± 0.2	7.5 ± 0.2	0.400	−0.06	7.3 ± 0.2	6.8 ± 0.1	0.005 *	−0.68
**TT**		10.5 ± 0.2	10.9 ± 0.2	0.552	0.34	10.7 ± 0.2	10.0 ± 0.2	0.010 *	−0.80
**LEFT**		107.6 ± 0.6	108.9 ± 0.7	0.003 *	0.53	107.9 ± 0.6	105.2 ± 0.6	<0.001 *	−1.02

**Notes.** d and nd indicate dominant and non-dominant limbs, respectively. DFt = dorsiflexion test; YBT = Y-balance test; SLCMJ = single-leg countermovement jump; LSI SLCMJ = limb simmetry Index countermovement jump; SLH = single-leg hop for distance; SH = side-hop test; SpJ = speed jump test; TT = agility T-test; LEFT = lower extremity functional test. SE = standard error. Statistical significance set at *p* < 0.050. * indicates statistically significant differences in post hoc comparisons.

## Data Availability

The original contributions presented in this study are included in the article. Further inquiries can be directed to the corresponding author(s).

## References

[1] Afonso J., Clemente F.M., Ribeiro J., Ferreira M., Fernandes R.J. (2020). Towards a de facto Nonlinear Periodization: Extending Nonlinearity from Programming to Periodizing. Sports.

[2] Liu G., Wang X., Xu Q. (2024). Microdosing Plyometric Training Enhances Jumping Performance, Reactive Strength Index, and Acceleration Among Youth Soccer Players: A Randomized Controlled Study Design. J. Sports Sci. Med..

[3] Pardos-Mainer E., Casajús J.A., Bishop C., Gonzalo-Skok O. (2020). Effects of Combined Strength and Power Training on Physical Performance and Interlimb Asymmetries in Adolescent Female Soccer Players. Int. J. Sports Physiol. Perform..

[4] Datson N., Drust B., Weston M., Jarman I.H., Lisboa P.J., Gregson W. (2017). Match Physical Performance of Elite Female Soccer Players During International Competition. J. Strength Cond. Res..

[5] Hameed I., Farooq N., Haq A., Aimen I., Shanley J. (2024). Role of strengthening exercises in management and prevention of overuse sports injuries of lower extremity: A systematic review. J. Sports Med. Phys. Fit..

[6] Gonzalo-Skok O., Tous-Fajardo J., Suarez-Arrones L., Arjol-Serrano J.L., Casajús J.A., Mendez-Villanueva A. (2017). Single-Leg Power Output and Between-Limbs Imbalances in Team-Sport Players: Unilateral Versus Bilateral Combined Resistance Training. Int. J. Sports Physiol. Perform..

[7] Waldén M., Krosshaug T., Bjørneboe J., Andersen T.E., Faul O., Hägglund M. (2015). Three distinct mechanisms predominate in non-contact anterior cruciate ligament injuries in male professional football players: A systematic video analysis of 39 cases. Br. J. Sports Med..

[8] Kuruganti U., Murphy T., Pardy T. (2010). Bilateral deficit phenomenon and the role of antagonist muscle activity during maximal isometric knee extensions in young, athletic men. Eur. J. Appl. Physiol..

[9] Gustavsson A., Neeter C., Thomeé P., Silbernagel K.G., Augustsson J., Thomeé R., Karlsson J. (2006). A test battery for evaluating hop performance in patients with an ACL injury and patients who have undergone ACL reconstruction. Knee Surg. Sports Traumatol. Arthrosc..

[10] Ramirez-Campillo R., Sanchez-Sanchez J., Gonzalo-Skok O., Rodríguez-Fernandez A., Carretero M., Nakamura F.Y. (2018). Specific Changes in Young Soccer Player’s Fitness After Traditional Bilateral vs. Unilateral Combined Strength and Plyometric Training. Front. Physiol..

[11] Zhang W., Chen X., Xu K., Xie H., Li D., Ding S., Sun J. (2023). Effect of unilateral training and bilateral training on physical performance: A meta-analysis. Front. Physiol..

[12] Gonzalo-Skok O., Sánchez-Sabaté J., Izquierdo-Lupón L., Sáez de Villarreal E. (2019). Influence of force-vector and force application plyometric training in young elite basketball players. Eur. J. Sport. Sci..

[13] McCurdy K., Conner C. (2003). Unilateral Support Resistance Training Incorporating the Hip and Knee. Strength Cond. J..

[14] Ide B.N., Silvatti A.P., Marocolo M., Santos C.P.C., Silva B.V.C., Oranchuk D.J., Mota G.R. (2022). Is There Any Non-functional Training? A Conceptual Review. Front. Sports Act. Living.

[15] Behm D., Colado J.C. (2012). The effectiveness of resistance training using unstable surfaces and devices for rehabilitation. Int. J. Sports Phys. Ther..

[16] Prieske O., Muehlbauer T., Krueger T., Kibele A., Behm D., Granacher U. (2015). Sex-specific effects of surface instability on drop jump and landing biomechanics. Int. J. Sports Med..

[17] Mehrpuya N., Moghadasi M. (2019). Effects of instability versus high-volume resistance training on thigh muscle cross-sectional area and hormonal adaptations. J. Phys. Act. Horm..

[18] Moisan G., Mainville C., Descarreaux M., Cantin V. (2022). Lower Limb Biomechanics During Drop-Jump Landings on Challenging Surfaces in Individuals With Chronic Ankle Instability. J. Athl. Train..

[19] Scinicarelli G., Trofenik M., Froböse I., Wilke C. (2021). The Reliability of Common Functional Performance Tests within an Experimental Test Battery for the Lower Extremities. Sports.

[20] Ferreira R.M., Martins P.N., Goncalves R.S. (2022). Effects of Self-myofascial Release Instruments on Performance and Recovery: An Umbrella Review. Int. J. Exerc. Sci..

[21] Boyle M., Verstegen M. (2012). Advances in Functional Training: Training Techniques for Coaches, Personal Trainers and Athletes.

[22] Moreno-Pérez V., Soler A., Ansa A., López-Samanes Á., Madruga-Parera M., Beato M., Romero-Rodríguez D. (2020). Acute and chronic effects of competition on ankle dorsiflexion ROM in professional football players. Eur. J. Sport. Sci..

[23] Calatayud J., Martin F., Gargallo P., García-Redondo J., Colado J.C., Marín P.J. (2015). The validity and reliability of a new instrumented device for measuring ankle dorsiflexion range of motion. Int. J. Sports Phys. Ther..

[24] García-Rubio J., Pino J., Olivares P.R., Ibáñez S.J. (2020). Validity and reliability of the WIMUTM inertial device for the assessment of joint angulations. Int. J. Environ. Res. Public Health.

[25] Fernández-González P., Koutsou A., Cuesta-Gómez A., Carratalá-Tejada M., Miangolarra-Page J.C., Molina-Rueda F. (2020). Reliability of Kinovea^®^ Software and Agreement with a Three-Dimensional Motion System for Gait Analysis in Healthy Subjects. Sensors.

[26] Nor Adnan N.M., Ab Patar M.N.A., Lee H., Yamamoto S.-I., Jong-Young L., Mahmud J. (2018). Biomechanical analysis using Kinovea for sports application. IOP Conf. Ser. Mater. Sci. Eng..

[27] Plisky P.J., Rauh M.J., Kaminski T.W., Underwood F.B. (2006). Star Excursion Balance Test as a predictor of lower extremity injury in high school basketball players. J. Orthop. Sports Phys. Ther..

[28] Young W. (1995). A simple method for evaluating the strength qualities of the leg extensor muscles and jumping abilities. Strength Cond. Coach.

[29] Marshall B.M., Moran K.A. (2013). Which drop jump technique is most effective at enhancing countermovement jump ability, “countermovement” drop jump or “bounce” drop jump?. J. Sports Sci..

[30] Holsgaard-Larsen A., Jensen C., Aagaard P. (2014). Subjective vs objective predictors of functional knee joint performance in anterior cruciate ligament-reconstructed patients—Do we need both?. Knee.

[31] Hildebrandt C., Müller L., Zisch B., Huber R., Fink C., Raschner C. (2015). Functional assessments for decision-making regarding return to sports following ACL reconstruction. Part I: Development of a new test battery. Knee Surg. Sports Traumatol. Arthrosc..

[32] Sassi R.H., Dardouri W., Yahmed M.H., Gmada N., Mahfoudhi M.E., Gharbi Z. (2009). Relative and absolute reliability of a modified agility T-test and its relationship with vertical jump and straight sprint. J. Strength Cond. Res..

[33] Brumitt J., Heiderscheit B.C., Manske R.C., Niemuth P.E., Rauh M.J. (2013). Lower extremity functional tests and risk of injury in division in collegiate athletes. Int. J. Sports Phys. Ther..

[34] Domínguez-Díez M., Castillo D., Raya-González J., Sánchez-Díaz S., Soto-Célix M., Rendo-Urteaga T., Lago-Rodríguez Á. (2021). Comparison of multidirectional jump performance and lower limb passive range of motion profile between soccer and basketball young players. PLoS ONE.

[35] Filter A., Olivares-Jabalera J., Dos’Santos T., Madruga M., Lozano J., Molina A., Santalla A., Requena B., Loturco I. (2023). High-intensity Actions in Elite Soccer: Current Status and Future Perspectives. Int. J. Sports Med..

[36] Chimera N.J., Swanik K.A., Swanik C.B., Straub S.J. (2004). Effects of plyometric training on muscle-activation strategies and performance in female athletes. J. Athl. Train..

[37] Afyon Y.A. (2014). Effect of core training on 16 year-old soccer players. Educ. Res. Rev..

[38] Kabaciński J., Gorwa J., Krakowiak W., Murawa M. (2025). Asymmetries of Force and Power During Single-Leg Counter Movement Jump in Young Adult Females and Males. Sensors.

[39] Michailidis Y., Stafylidis A., Mandroukas A., Kyranoudis A.E., Antoniou G., Kollias R., Kanaras V., Bamplekis C., Vardakis L., Semaltianou E. (2025). Correlation of the Asymmetry Index from the Single-Leg Countermovement Jump with the Asymmetry Index from Isokinetic Strength in Elite Youth Football Players. Appl. Sci..

[40] Taylor K.-L., Cronin J., Gill N.D., Chapman D.W., Sheppard J. (2010). Sources of Variability in Iso-Inertial Jump Assessments. Int. J. Sports Physiol. Perform..

[41] Jakobsen L.S., Madeleine P., Pavailler S., Lefebvre F., Giandolini M. (2022). The effects of unstable surface conditions on lower limb biomechanical parameters during running. J. Biomech..

[42] Mohammadi H., Ghaffari R., Kazemi A., Behm D.G., Hosseinzadeh M. (2024). Evaluation of the lower extremity functional test to predict lower limb injuries in professional male footballers. Sci. Rep..

[43] Polzer H., Kanz K.G., Prall W.C., Haasters F., Ockert B., Mutschler W., Grote S. (2012). Diagnosis and treatment of acute ankle injuries: Development of an evidence-based algorithm. Orthop. Rev..

[44] Jouira G., Alexe D.I., Tohănean D.I., Alexe C.I., Tomozei R.A., Sahli S. (2024). The Relationship between Dynamic Balance, Jumping Ability, and Agility with 100 m Sprinting Performance in Athletes with Intellectual Disabilities. Sports.

[45] Pietraszewski P., Gołaś A., Zając A., Maćkała K., Krzysztofik M. (2025). The Acute Effects of Combined Isometric and Plyometric Conditioning Activities on Sprint Acceleration and Jump Performance in Elite Junior Sprinters. Appl. Sci..

